# Significantly Elevated Serum Lipase in Pregnancy with Nausea and Vomiting: Acute Pancreatitis or Hyperemesis Gravidarum?

**DOI:** 10.1155/2015/359239

**Published:** 2015-02-02

**Authors:** Amanda Johnson, Bethany Cluskey, Nina Hooshvar, Daphne Tice, Courtney Devin, Elaine Kao, Suhalia Nawabi, Steven Jones, Lihua Zhang, Chi Dola

**Affiliations:** ^1^Section of Maternal-Fetal Medicine, Department of Obstetrics and Gynecology, SL-11, Tulane School of Medicine, 1430 Tulane Avenue, New Orleans, LA 70112, USA; ^2^Department of General Surgery, Tulane School of Medicine, 1430 Tulane Avenue, New Orleans, LA 70112, USA; ^3^Tulane-Lakeside Hospital, 4700 S I 10 W. Service Road, Metairie, LA 70001, USA; ^4^Department of Anesthesiology, Tulane School of Medicine, 1430 Tulane Avenue, New Orleans, LA 70112, USA

## Abstract

Hyperemesis gravidarum is a severe manifestation of nausea and vomiting of pregnancy and it is associated with weight loss and metabolic abnormalities. It is known that abnormal laboratory values, including mildly elevated serum lipase level, could be associated with hyperemesis gravidarum. However, in this case report details of two women with hyperemesis gravidarum but with significantly elevated serum lipase levels were discussed. These patients presented with severe nausea and vomiting but without abdominal pain. They were found to have severely elevated lipase levels over 1,000 units/liter. In the absence of other findings of pancreatitis, they were treated with conservative measures for hyperemesis gravidarum, with eventual resolution to normal lipase levels. Although significantly elevated lipase level in pregnant patients with nausea and vomiting is a concern for acute pancreatitis, these two cases of significantly elevated serum lipase without other clinical findings of pancreatitis led to this report that serum lipase could be quite elevated in hyperemesis gravidarum and that it might not be an accurate biochemical marker for acute pancreatitis. Imaging studies are thus necessary to establish the diagnosis of acute pancreatitis.

## 1. Introduction

Nausea and vomiting of pregnancy is the most common medical condition affecting pregnancy and is present in up to 85% of gestations [1, 2]. Symptoms usually begin between 4 and 6 weeks of gestation, peak between 8 and 12 weeks, and resolve by 20 weeks. Severity ranges from mild symptoms of nausea and vomiting to severe incapacitation, or hyperemesis gravidarum. It is a diagnosis of exclusion that features severe and persistent vomiting, weight loss of more than 5 percent of prepregnancy weight, dehydration, electrolyte imbalances, and nutritional deficiencies. The incidence ranges from 0.5 to 2% of pregnancies [1]. It represents the most common cause of hospitalization in the first trimester and is the second most common cause of hospitalization in pregnancy overall [1].

The etiology of hyperemesis gravidarum is likely multifactorial, involving genetic, endocrine, and neurobehavioral factors [3–5]. Risk factors include increased placental mass, as seen with multiple gestations or trophoblastic disease, a personal or family history of hyperemesis gravidarum, a history of motion sickness or migraines, and carrying a female fetus [1, 4]. Hyperemesis gravidarum is a diagnosis of exclusion, with other potential causes of nausea and vomiting in pregnancy including multiple gestation, hydatidiform mole, preeclampsia, gastroenteritis, dyspepsia, gastroparesis, gastroesophageal reflux disease, cholecystitis, pancreatitis, migraines, malignancy of the central nervous system, pseudotumor cerebri, hyperthyroidism, uremia, Addison's disease, diabetic ketoacidosis, pyelonephritis, and drug toxicity [1]. Laboratory studies are often used to aid in the differential diagnosis. Common findings of hyperemesis gravidarum include elevated liver enzymes (<300 units/L), elevated serum bilirubin (<4 mg/dL), elevated serum amylase or lipase, decreased serum thyroid stimulating hormone, increased serum free thyroxine, and elevated specific gravity and/or ketonuria on urinalysis [1, 6]. In this paper, we present two cases of hyperemesis gravidarum but with significantly elevated serum lipase levels and without signs of pancreatitis.

## 2. Case Report

In the first case, a 29-year-old gravida-2-para-1-0-0-1 African American female was transferred to our hospital from an outside facility at 16-week gestation with a complaint of nausea, vomiting, and inability to tolerate oral intake for one day. Her pregnancy had been complicated by hyperemesis gravidarum and chronic hypertension. At the time of presentation, physical exam revealed normal vital signs and a soft abdomen with mild tenderness in the suprapubic area. Laboratory studies were significant for a white blood cell (WBC) count of 18.6, potassium of 2.7 millimoles/liter (mmol/L), aspartate aminotransferase (AST) of 94 units/L, alanine aminotransferase (ALT) of 189 units/L, amylase of 129 units/L, and >160 ketones on urinalysis. The patient was admitted to the hospital with presumptive diagnosis of hyperemesis gravidarum and given intravenous fluid hydration with potassium replacement and antiemetics, including vitamin B6 25 milligrams (mg) administered intravenously (IV) every 8 hours, ondansetron 4 mg IV every 6 hours as needed (prn), and promethazine 12.5 mg IV every 6 hours prn. By hospital day 2, her emesis had improved and her abdominal pain had resolved. Laboratory studies at this time showed an amylase of 179 units/L and lipase of 1,324 units/L, while AST and ALT had decreased to 66 units/L and 164 units/L, respectively. Abdominal ultrasound was performed and revealed biliary sludge without signs of cholelithiasis or cholecystitis. General surgery and maternal-fetal medicine were consulted for elevated amylase and lipase in the setting of nausea and vomiting in a pregnant woman. In the setting of elevated lipase, conservative measures with no oral intake were recommended initially due to concern for the possible diagnosis of acute pancreatitis. On hospital day 8, her nausea had resolved completely, and the patient was started on a clear liquid diet. Despite resolution of her symptoms of nausea and vomiting, her lipase continued to increase to a peak level of 2,739 units/L. Repeat abdominal ultrasound again demonstrated no evidence of cholelithiasis or pancreatic abnormalities. On hospital day 14, the patient was tolerating a soft mechanical diet. Her lipase had decreased to 1,025 units/L. As patient improved significantly despite elevated lipase level, it was believed that the elevated lipase was associated with hyperemesis gravidarum rather than acute pancreatitis. She was discharged home at that time in stable condition. Follow-up in clinic revealed normalization of her lipase after one month. This remained normal for the rest of pregnancy and she had an uncomplicated delivery at term.

In the second case, a 22-year-old gravida-2-para-0-1-0-1 African American female presented to the hospital at 9-week gestation with a one-week history of nausea and vomiting unresponsive to oral antiemetics, including sublingual ondansetron and rectal promethazine. She had been unable to tolerate oral intake for two days prior to presentation. At the time of presentation, physical exam demonstrated normal vital signs and a nontender abdomen. Laboratory studies at the time of admission revealed a WBC count of 13.3, AST of 35 units/L, ALT of 42 units/L, amylase of 137 units/L, and lipase of 436 units/L. The patient was admitted to the hospital on the maternal-fetal medicine service with the presumptive diagnosis of hyperemesis gravidarum and was given intravenous fluid hydration and antiemetics, including vitamin B6 10 mg IV every 6 hours, ondansetron 8 mg IV every 6 hours prn, promethazine 12.5 mg IV every 4 hours prn, and scopolamine 1.5 mg transdermal patch every 72 hours. On hospital day 3, serum amylase had increased to 212 units/L and lipase had increased to 1,022 units/L. The patient continued to experience severe emesis. Abdominal ultrasound exam demonstrated a normal appearing gallbladder without evidence of cholelithiasis, cholecystitis, pancreatitis, or peripancreatic fluid. General surgery was consulted for significantly elevated lipase level and recommended conservative management, with no oral intake. While the patient's nausea continued, she had minimal epigastric abdominal pain. Serum lipase level decreased to 594 units/L on hospital day 5. Her lipid panel was within normal limits. Due to the patient's continued vomiting, she was started on total parenteral nutrition (TPN) on hospital day 7. In spite of conservative therapy with no oral intake and IV antiemetics, the patient continued to have significant nausea and vomiting. Thus, a three-day course of steroid therapy with methylprednisolone 2 mg every 8 hours was started on hospital day 9. On hospital day 9, she was noted to have continued nausea and subjective worsening of her abdominal pain. However, she did not have abdominal distention, guarding, rebound tenderness, or fever. Her lipase increased to 1,448 units/L. Her lipase remained elevated (>700 units/L) over the next six days. She was continued on TPN. On hospital day 18, lipase had decreased to 662 units/L. The patient continued to have nausea, but emesis and abdominal pain had resolved. She was discharged home on TPN and an ondansetron pump, which administered 1 mg of ondansetron subcutaneously over 1 hour continuously, for a total dose of 24 mg per day. During follow-up as an outpatient, her lipase returned to a normal level at two weeks after discharge.

This level remained normal for the rest of pregnancy, and the patient had a delivery at 36-week gestation, complicated by severe preeclampsia.

At our hospital, the serum lipase level was quantitatively measured in human serum and plasma on the dimension clinical chemistry system using the LIPL method (SIEMENS). In summary, the LIPL method uses the pancreatic enzyme lipase to hydrolyze the substrate 1-2-O-dilauryl-rac-glycero-3-glutaric acid-(6′-methylresorufin)-ester in the presence of colipase, bile salt, and calcium. This reaction produces 1-2-O-dilauryl-rac-glycerol + glutaric-6′-methylresorufin-ester, which is unstable. It then breaks down and produces free methylresorufin. The quantity of methylresorufin being produced is measured photometrically and is in proportion to the activity of lipase present in the sample [7].

## 3. Discussion

The two cases of hyperemesis gravidarum described involve significantly elevated lipase levels. In cases of nausea and vomiting of pregnancy with associated elevations in serum lipase, the diagnosis of pancreatitis must be excluded. Pancreatitis occurs in approximately one out of 3,000 pregnancies [8, 9]. Causes include cholelithiasis, heavy alcohol use, hypertriglyceridemia, and metabolic disorders [8, 10]. Pancreatitis typically presents with severe and persistent abdominal pain in the midepigastric or left upper quadrant region with radiation to the back, chest, flanks, or lower abdomen. Physical exam findings include abdominal tenderness fever, tachycardia, and jaundice. Diagnostic criteria for pancreatitis in pregnancy include the presence of typical abdominal pain, elevated serum amylase or lipase to levels greater than three times normal serum values, and confirmatory findings of pancreatitis on abdominal imaging [8, 11]. In contrast to this presentation, patients with hyperemesis gravidarum usually present with nausea and vomiting as their main complaints. Abdominal pain is not a common presenting symptom, and significant abdominal pain and fever are not usually seen on physical exam [1].

Serum lipase has a better sensitivity (94%) and specificity (96%) for diagnosing acute pancreatitis when compared to serum amylase (83% and 88%, resp.) [10]. While liver enzyme levels may be elevated in those with pancreatitis due to cholelithiasis or alcohol use, these levels alone are not reliable for diagnosing acute pancreatitis. A common cause of pancreatitis in pregnancy is lipid-induced pancreatitis from elevated triglyceride levels [2, 4]. In a normal pregnancy, triglyceride levels may increase up to 300 mg/dL. Levels greater than 750 mg/dL increase the risk for pancreatitis [8]. Therefore, a fasting lipid panel should be performed in patients with suspected pancreatitis. While CT scan is the imaging modality of choice to confirm the diagnosis of pancreatitis in the nonpregnant population, an abdominal ultrasound exam is the best modality in pregnant patients [9]. It allows for evaluation of pancreatic inflammation as well as gallstones.

In the two cases described above, the patients had features of pancreatitis, including severely elevated lipase levels, nausea, and vomiting. However, their main complaints of severe nausea and vomiting were consistent with the diagnosis of hyperemesis gravidarum. While pancreatitis was a consideration in the differential diagnosis, especially in light of their significantly elevated lipase levels, they did not meet other criteria for this diagnosis. Neither patient had the severe abdominal pain typical of pancreatitis or imaging findings on ultrasound consistent with pancreatitis.

Pancreatitis is treated conservatively with bowel rest, fluid hydration, and pain medication [9]. Patients may also be treated with endoscopic retrograde cholangiopancreatography, although most attempts are made to decrease surgical interventions in patients that present outside the second trimester [8, 9]. In the patients described above, conservative treatments for hyperemesis gravidarum, including intravenous fluid hydration and antiemetics, were employed. While a consideration could be given to bowel rest in light of the severely elevated lipase levels, the patients were allowed to consume a normal diet as they began to feel hungry. Over the next several weeks, while the patients were followed on an outpatient basis, their lipase levels again returned to normal without further intervention.

Significantly elevated lipase level in pregnant patients with nausea and vomiting requires a work-up for acute pancreatitis. However, these two cases of significantly elevated serum lipase without other clinical findings of pancreatitis led to this report that serum lipase could be quite elevated in hyperemesis gravidarum and that serum lipase level might not be an accurate biochemical marker for acute pancreatitis. A review of available literature found three abstracts in the gastroenterology literature describing elevated amylase and lipase associated with hyperemesis gravidarum [6, 12, 13]. The elevations of amylase and lipase were often mild, as compared to the severe elevations in our cases. The lipase levels range from 171 units/L in one study [6] up to 6-fold increase over the upper limit of normal in another study [13]. It is important to rule out other causes of nausea and vomiting during pregnancy, including acute pancreatitis. However, in patients with presentations that are not consistent with pancreatitis, it is difficult to understand why lipase levels would be significantly elevated. We question whether this elevation of lipase is another physiologic change of pregnancy or whether nausea and vomiting triggers the production of lipase from another source besides the pancreas. A case series described by Frank and Gottlieb discussed nonpancreatic sources of lipase [14]. They cited that certain organs of the gastrointestinal tract, such as the tongue, esophagus, gastroesophageal junction, stomach, duodenum, small bowel, and liver, contain enzymes with lipolytic activity [14]. Therefore disorders from obstructive and inflammatory bowels, intestinal infarction, duodenal ulcer, liver disease, and abdominal trauma can trigger production of lipase enzymes from these nonpancreatic sources [14]. Diabetic ketoacidosis, asymptomatic chronic alcoholism, and renal insufficiency were also reported to cause isolated elevation of lipase [14]. Thus, it appears that several disease processes aside from pancreatitis can cause isolated elevation of serum lipase. We now add hyperemesis gravidarum as another nonpancreatic cause of significantly elevated serum lipase. Could it be that hyperemesis gravidarum affects the organs of the gastrointestinal tract, thus triggering the production of lipase from these nonpancreatic organs? Further research is much needed to understand the physiologic mechanism of high serum lipase level in hyperemesis gravidarum and its clinical significance.

## References

[1] American College of Obstetricians and Gynecologists (ACOG) (2004). Nausea and vomiting of pregnancy. *Obstetrics and Gynecology*.

[2] Einarson T. R., Piwko C., Koren G. (2013). Prevalence of nausea and vomiting of pregnancy in the USA: a meta-analysis. *Journal of Population Therapeutics and Clinical Pharmacology*.

[3] Katon W. J., Ries R. K., Bokan J. A., Kleinman A. (1980). Hyperemesis gravidarum: a biopsychosocial perspective. *International Journal of Psychiatry in Medicine*.

[4] Kramer J., Bowen A., Stewart N., Muhajarine N. (2013). Nausea and vomiting of pregnancy: prevalence, severity and relation to psychosocial health. *MCN The American Journal of Maternal/Child Nursing*.

[5] Sanu O., Lamont R. F. (2011). Hyperemesis gravidarum: pathogenesis and the use of antiemetic agents. *Expert Opinion on Pharmacotherapy*.

[6] Carballo M., Kotwal V., Demetria M., Attar B. A case of elevated lipase: beyond the pancreas.

[7] Siemens Healthcare Diagnostics (2013). *Dimension Clinical Chemistry System on LIPL Method [Package Insert]*.

[8] Angelini D. J. (2003). Obstetric triage revisited: update on non-obstetric surgical conditions in pregnancy. *Journal of Midwifery & Women's Health*.

[9] Pitchumoni C. S., Yegneswaran B. (2009). Acute pancreatitis in pregnancy. *World Journal of Gastroenterology*.

[10] Ducarme G., Maire F., Chatel P., Luton D., Hammel P. (2014). Acute pancreatitis during pregnancy: a review. *Journal of Perinatology*.

[11] Longo D. L., Fauci A. S., Kasper D. L., Hauser S. L., Jameson J. L., Loscalzo J. L. (2012). *Harrison's Principles of Internal Medicine*.

[12] Firth M., Burton F. R., Prather C. M., Artal R. (2003). Elevated amylase & lipase: a frequent finding in hyperemesis gravidarum (HG). *Gastroenterology*.

[13] Im G. Y., Squillace B. A., Sidhu-Buonocore S., Grendell J. H. (2010). S1376 Elevated amylase and lipase levels in hyperemesis gravidarum: accurate markers of acute pancreatitis?. *Gastroenterology*.

[14] Frank B., Gottlieb K. (1999). Amylase normal, lipase elevated: is it pancreatitis?: a case series and review of the literature. *The American Journal of Gastroenterology*.

